# The choice of tree prior and molecular clock does not substantially affect phylogenetic inferences of diversification rates

**DOI:** 10.7717/peerj.6334

**Published:** 2019-03-13

**Authors:** Brice A.J. Sarver, Matthew W. Pennell, Joseph W. Brown, Sara Keeble, Kayla M. Hardwick, Jack Sullivan, Luke J. Harmon

**Affiliations:** 1 Department of Biological Sciences and Institute for Bioinformatics and Evolutionary Studies, University of Idaho, Moscow, ID, USA; 2 Department of Zoology and Biodiversity Research Centre, University of British Columbia, Vancouver, BC, Canada; 3 Department of Animal and Plant Sciences, University of Sheffield, Sheffield, UK; 4 Department of Molecular and Computational Biology, University of Southern California, Los Angeles, CA, USA

**Keywords:** Phylogenetic comparative methods, Birth–death process, Diversification rates, Molecular clock, Yule process

## Abstract

Comparative methods allow researchers to make inferences about evolutionary processes and patterns from phylogenetic trees. In Bayesian phylogenetics, estimating a phylogeny requires specifying priors on parameters characterizing the branching process and rates of substitution among lineages, in addition to others. Accordingly, characterizing the effect of prior selection on phylogenies is an active area of research. The choice of priors may systematically bias phylogenetic reconstruction and, subsequently, affect conclusions drawn from the resulting phylogeny. Here, we focus on the impact of priors in Bayesian phylogenetic inference and evaluate how they affect the estimation of parameters in macroevolutionary models of lineage diversification. Specifically, we simulate trees under combinations of tree priors and molecular clocks, simulate sequence data, estimate trees, and estimate diversification parameters (e.g., speciation and extinction rates) from these trees. When substitution rate heterogeneity is large, diversification rate estimates deviate substantially from those estimated under the simulation conditions when not captured by an appropriate choice of relaxed molecular clock. However, in general, we find that the choice of tree prior and molecular clock has relatively little impact on the estimation of diversification rates insofar as the sequence data are sufficiently informative and substitution rate heterogeneity among lineages is low-to-moderate.

## Introduction

Statistical comparative methods use phylogenetic trees to gain insight into macroevolutionary patterns and processes (Felsenstein, 1985; Harvey & Pagel, 1991; O’Meara, 2012; Rabosky, 2016; Harmon, 2018). Branch lengths and node ages provide information about the rate of lineage accumulation through time (Nee, May & Harvey, 1994; Nee, 2006; Ricklefs, 2007; Pyron & Burbrink, 2013) and are instrumental in characterizing the underlying processes generating global patterns of biodiversity (Schluter & Pennell, 2017). A typical workflow uses a point estimate of a phylogenetic tree or a distribution of trees to estimate macroevolutionary parameters, such as the rate of lineage accumulation (speciation) or extinction, which are often compared across groups to provide insight into diversification rates and the tempo of evolution (Nee, Mooers & Harvey, 1992; Magallón & Sanderson, 2001; Alfaro et al., 2009; Rabosky, 2014). However, parameter estimates are dependent on the tree from which they are inferred (Felsenstein, 1985). Most inference procedures assume that a tree is estimated without error, but, because branch lengths are fundamental to estimates of diversification parameters, uncertain phylogenies can be expected to yield uncertain estimates. Several studies have focused on the causes of parameter misestimation when fitting diversification models to trees (Nee, May & Harvey, 1994; Barraclough & Nee, 2001; Revell, Harmon & Glor, 2005; Cusimano & Renner, 2010; Rabosky, 2010; Moore et al., 2016), and a handful have evaluated uncertainty in phylogenetic estimation explicitly in the context of estimating diversification rates from phylogenies under specific simulation conditions (Revell, Harmon & Glor, 2005; Wertheim & Sanderson, 2011; Marin & Hedges, 2018).

Theoretical advances have expanded the scope of phylogenetic comparative methods for studying diversification. Historically, models only assumed a constant rate of lineage diversification or extinction (Nee, May & Harvey, 1994). More modern approaches utilize phylogenies to determine where and/or when shifts in the rates of speciation and extinction take place (see Pyron & Burbrink, 2013) or estimate rates that depend on species’ traits (Maddison, Midford & Otto, 2007; FitzJohn, Maddison & Otto, 2009; FitzJohn, 2010).

It has been shown that phylogenetic uncertainty and error in tree estimation can directly impact the results of diversification studies. For example, Revell, Harmon & Glor (2005) demonstrated that underparameterization of the model of nucleotide sequence evolution as part of the process of phylogenetic estimation can produce apparent slowdowns in the rate of diversification as quantified by Pybus & Harvey’s (2000) gamma statistic. Additionally, errors in branch lengths (Wertheim & Sanderson, 2011) and biased taxonomic sampling can both affect estimates (Höhna, 2014). Taken together, these studies suggest that phylogenetic error can affect the estimation of diversification rates.

Bayesian methods of phylogenetic inference produce posterior distributions of trees, and, therefore, diversification rates can be estimated across such distributions to quantify uncertainty. The use of Bayesian approaches in phylogenetics has increased in recent years due in part to the availability of software, including Bayesian Evolutionary Analysis by Sampling Trees (BEAST) (Drummond et al., 2012) and MrBayes (Ronquist et al., 2012). BEAST is a Java application that has seen widespread use in the phylogenetics community due to its ease-of-use, intuitive graphical user interface, and implementation of a number of phylogenetic and population genetic models. BEAST may also be run from the command line and can leverage GPU hardware, facilitating phylogeny reconstruction on high-performance computing architectures. Users can specify an analysis by passing options from the command line or through a GUI to a bundled application, BEAUti, which produces the XML input file required for BEAST. In a typical analysis, this XML defines models of sequence evolution, a choice of branching model (i.e., tree prior), and a choice of molecular clock, among other possible configurations.

The impact that the choice of priors governing the molecular clock and branching process (or “tree prior”) in molecular phylogenetics is an active area of research. Commonly used tree priors for inference among multiple species are the Yule (1925) and birth–death (BD; Kendall, 1948; Nee, May & Harvey, 1994; Gernhard, 2008; Stadler, 2013) models, whereas coalescent-based priors are suitable for phylogenetic and population genetic studies within a single species (Kingman, 1982; see Drummond et al., 2002, 2005). Here, we focus on the Yule and BD models. The Yule model is the simplest of a group of continuous-time branching processes; it has one parameter, λ, the instantaneous per-lineage rate of speciation, that is constant across the tree. The BD model is also a continuous-time process but includes a probability that a lineage will go extinct (and, therefore, leave no descendants). This model has two parameters, λ and μ, the instantaneous per-lineage rates of speciation and extinction, both of which are constant across the tree in their original characterizations. In practice, many approaches re-parameterize the model using *r* = (λ − μ) and ε = (μ/λ), the net diversification rate and relative extinction rate, respectively. In general, estimates of *r* have greater precision than ε (Nee et al., 1994; Nee, May & Harvey, 1994; FitzJohn, Maddison & Otto, 2009). Upon selecting these tree priors when using BEAST, a prior distribution (technically, hyperpriors in a hierarchical Bayesian context) must be specified on λ or on *r* and ε for Yule or BD, respectively.

Diversification rates can be estimated from phylogenies using likelihood-based approaches that rely on branching times (see Stadler, 2013). As a result, it is reasonable to assume that different branching models could have an impact on diversification rate estimates by virtue of altering branch lengths. Several studies have explored the impact of the tree prior on the resulting phylogenetic estimates. As part of an investigation of relaxed clock models, Ho et al. (2005) identified an impact of the choice of birth and death rate upper bounds in concert with the fraction of lineages sampled, particularly with respect to internal branches. Furthermore, in Ritchie, Lo & Ho (2017), the authors explore the impact of Yule and BD (and, additionally, coalescent) tree priors in the context of the multispecies coalescent to determine whether prior misspecification has an impact on phylogenetic accuracy. Through simulations and applications to empirical datasets, they concluded that phylogenies are not substantially affected by tree prior misspecification. However, node times may be influenced by the choice of prior in combination with among- and within-lineage sampling. Additionally, Brown & Yang (2010) found that, for shallow phylogenies, nodes depths are generally robust to the choice of prior. However, they concluded that a Dirichlet prior, in contrast to BD, produces more reasonable estimates as the depth of the phylogeny increases.

In addition to priors for branching process parameters, Bayesian phylogenetic analysis also requires the specification of a particular model for rates of evolution across the tree. For example, BEAST gives users the choice of using a strict (global) molecular clock or an uncorrelated log-normal (UCLN) relaxed molecular clock, among other flavors of local clocks (Drummond et al., 2012). The strict clock assumes a constant, global rate of sequence evolution across the tree (Zuckerkandl & Pauling, 1962), while the UCLN relaxed clock assumes branch-specific rates are drawn from a discretized log-normal distribution independently for every branch in the tree (Drummond et al., 2006). Hyperpriors are placed on the mean rate of evolution for the strict clock and the mean and standard deviation of the log-normal distribution for the UCLN relaxed clock. As the name implies, the UCLN molecular clock assumes that rates of evolution are not correlated among branches. This is in contrast with approaches commonly used to scale phylogenies after estimation, such as the penalized likelihood approach implemented in r8s (Sanderson, 2003) or treePL (Smith & O’Meara, 2012), which may inappropriately infer similar rates among closely-related lineages. However, the effect of selecting uncorrelated models over autocorrelated models may not always be clear and warrants further consideration (Ho et al., 2005; Lepage et al., 2007).

As with tree priors, the choice of molecular clock could also be expected to affect diversification rate estimates a priori by impacting branch lengths. Lepage et al. (2007) compare several clock models and show that clock choice can impact the estimates of divergence times. Furthermore, they find that clock misspecification can have a larger impact than the choice of branching prior. Previous work has also shown that relaxed clock models produce reasonable estimates of rates when substantial rate variation is not observed (Ho et al., 2005).

From the results outlined above, there is reason to believe that the choice of priors can affect the estimation of diversification parameters by virtue of altering the distribution of branch lengths. This has been explored specifically in several studies. For example, the effects of tree reconstruction on diversification rate estimates were studied by Wertheim & Sanderson (2011). This study focused on trees generated only under a Yule process with a range of λ values. The authors simulated sequences under a simple model of sequence evolution (HKY85), and trees were estimated using BEAST assuming a strict clock and narrow prior or range of prior widths on the root age. Their study assessed the impact of sequence length and nodal calibrations on estimating posterior distributions of λ, and they found that increasing sequence length leads, as expected, to narrower 95% highest posterior density credible intervals of speciation rates. Additionally, broader calibration priors were shown to increase posterior widths of these estimates. It is plausible that forcing estimation of a tree under a particular branching process (such as a Yule process) may impact estimates if the true generating process was different (such as a BD process); this could systematically affect diversification parameter estimates.

Since branch lengths play an important part in estimating diversification parameters, it is also the case that a mismatch of clock models could similarly affect results. Whereas previous work describes a relationship between parameter estimation and misspecification of the model of nucleotide sequence evolution during phylogenetic estimation (Revell, Harmon & Glor, 2005), as well as sequence length and nodal calibrations (Wertheim & Sanderson, 2011), no studies to our knowledge have directly focused on the impact of tree priors and choice of molecular clocks combined (but see Condamine et al., 2015 for comparisons among Yule and BD priors using an empirical dataset). Additionally, a recent study by Duchêne, Hua & Bromham (2017) emphasizes the importance of appropriately accommodating among-lineage molecular rate variation when inferring diversification rates, both of which may be correlated through underlying evolutionary processes. This study simulated datasets under a variety of diversification conditions with a constant background extinction rate and stressed the importance of accurately capturing variable substitution rates as part of reconstructing the phylogeny.

Following a Bayesian statistical philosophy, ideally priors should be selected which reflect a priori knowledge about the data being explored. However, such knowledge may not always be available for each study of interest, especially in non-model systems. It may be possible to use reasonable defaults as selected by an application of choice; however, there is no guarantee that results will always be accurate. One way to tackle this may be to select uninformative (i.e., broad) priors under the assumption that there is enough signal in the data to produce reliable estimates. This can be assessed by performing a parallel analysis sampling only from prior distributions and comparing results to real data or through posterior predictive simulations (Gelman et al., 1995; Huelsenbeck et al., 2001). An alternative approach may be to select the most parameter-rich models with the hope that more complex patterns in the data will be captured and modeled appropriately. However, as the number of parameters increases, issues could arise with overfitting and identifiability, necessitating the use of model selection for comparison of fit. Here, this study is motivated by the observation that phylogenetics, sensu lato, is complex, and the uninitiated may resort to using defaults assigned in tutorials or documentation. At least one other study has explicitly mentioned this (Condamine et al., 2015), referencing selection of a Yule tree prior as suggested in an early BEAST tutorial.

In light of these concerns, we are interested in exploring the choice of tree and molecular clock priors as part of a simulation study conducted using BEAST with choices that researchers may naturally select when interrogating their data. Since we already know that misestimation of the absolute root age of the tree can have dramatic effects on rate estimates, we focus specifically on the effect of priors on relative branch lengths of trees. We quantify the effect of tree prior and clock misspecification on subsequent parameter estimates for diversification models. To accomplish this, we simulate phylogenetic trees and sequence data under a range of combinations of tree priors and molecular clock models. We then re-estimate trees and use these reconstructed trees to calculate maximum likelihood estimates of diversification rate parameters. We compare these estimates to ones from the original trees to evaluate whether or not priors and clock models contribute to error in estimating diversification rates.

## Materials and Methods

**Figure 1:**
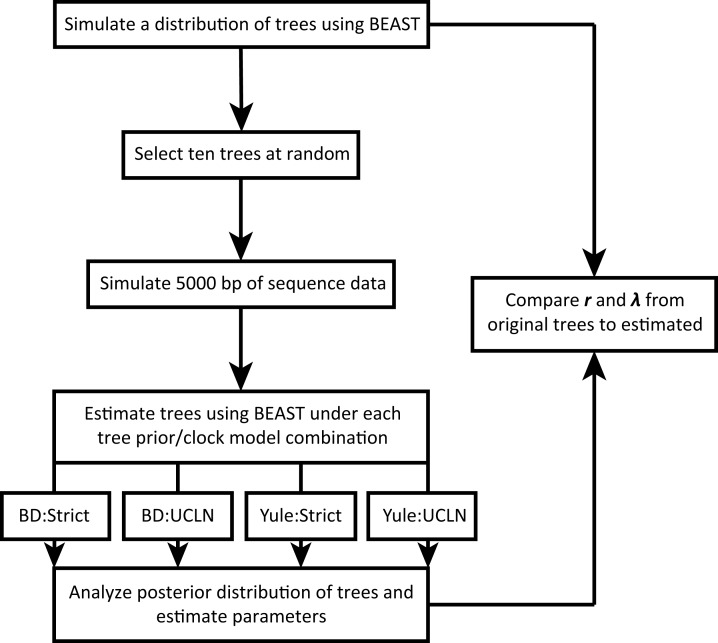
Simulation workflow. λ is the instantaneous speciation rate, and *r* is the net diversification rate. Both are estimated for each set of simulation conditions.

We take advantage of existing applications to simulate trees under a variety of conditions, simulate nucleotide sequence data on these trees, estimate a tree from the nucleotide data, and estimate diversification rates. The workflow is illustrated in Fig. 1. All scripts are written in the R programming language (R Development Core Team, 2018) and are available on GitHub (https://github.com/bricesarver/prior_simulation_study).

### Generation of initial distributions of trees

We simulated trees of two sizes, 25 and 100 taxa, both with a tree depth of five arbitrary time units. We simulated initial trees using BEAST v1.7.5 with XML input files generated using BEAUti v1.7.5 (Drummond et al., 2012). DNA sequence data were simulated using these trees with SeqGen v1.3.2 (Rambaut & Grassly, 1997).

The simulation process itself consisted of two steps. First, a tree prior was selected for each round of simulations, either Yule or BD. In order to avoid improbable combinations of parameters such that tree shapes were non-randomly sampled (Pennell, Sarver & Harmon, 2012), initial parameter values for *N*_t_ were fixed and *r* calculated using the expectation relating the net diversification rate, the number of taxa, and the tree height: E[*N_t_*] = *N*_0_e^*rt*^, where *N_t_* is the number of taxa at *t*, *N*_0_ is the initial number of taxa (2 in this case), *r* is the net diversification rate (λ − μ), and *t* is the height of the tree (Nee, 2006). Therefore, when *N_t_* = 25, *r* = 0.5051, and when *N_t_* = 100, *r* = 0.7824, both with a tree height of 5. For BD cases, ε was fixed at 0.5.

BEAST requires the specification of a type of molecular clock. For the strict case, the prior on the clock rate was fixed to a log-normal distribution with a mean of 0.01 and a standard deviation of 0.5. For the UCLN case, the prior on the mean of the distribution was of the form U(0.0050, 0.015), and the prior on the standard deviation of the distribution was set to either U(0.17, 0.18), U(0.25, 1), or U(0.25, 1.75). Together, these simulations correspond to a low, medium, and high amount of among-lineage substitution rate heterogeneity.

We then generated a distribution of trees under these conditions using BEAST, sampling only from the priors. To “fix” a parameter, such as root height, to a given value, a normal prior was used with a mean equal to the value and a standard deviation of 0.00001. This prevented BEAST failures using a prior with hard boundary conditions.

### Simulation of nucleotide datasets

For each set of parameter values, we generated a posterior distribution of 10,001 phylograms by sampling from the prior. A total of 10 trees were selected at random without replacement. 5,000 bp of sequence data (see Wertheim & Sanderson, 2011) were simulated under a GTR+Γ model of nucleotide sequence evolution with parameters estimated in Weisrock, Harmon & Larson (2005) for nuclear rRNA (π_A_: 0.1978, π_C_: 0.2874, π_G_: 0.3403, π_T_: 0.1835; r_AC_: 1.6493, r_AG_: 2.9172, r_AT_: 0.3969, r_CG_: 0.9164 r_CT_: 8.4170, r_GT_: 1.0; α: 2.3592). Sequences were simulated using Seq-Gen v1.3.5 (Rambaut & Grassly, 1997) with randomly generated seeds. Additionally, we simulated datasets of two additional sizes, 2,500 and 10,000 bp, for the 100-taxa, BD:UCLN case to assess the impact of sequence length on parameter estimates. We expect the accuracy of parameter estimates to improve as the amount of sequence data increases owing to more accurate estimation of branch lengths.

### Estimation under tree prior and clock combinations

The resulting NEXUS data files were processed using BEASTifier v1.0 (Brown, 2014). BEASTifier takes a list of NEXUS files and generates BEAST XML input files under conditions specified in a configuration file. Each combination of tree priors and clock types was used for each dataset. For example, the sequences generated using a 100-taxa tree that is simulated under a Yule tree prior and strict molecular clock ultimately produced four XML files for analysis: the condition matching the simulation conditions (i.e., a posterior distribution of trees using a Yule tree prior and a strict clock (1)) and all mismatch conditions (i.e., a posterior distribution of trees using a Yule tree prior and a UCLN clock (2), a BD tree prior and a strict clock (3), and a BD prior and UCLN clock (4)). Each file was then processed using BEAST v1.7.5 (Drummond et al., 2012). Chains were run for 25,000,000 generations (standard analyses) or 50,000,000 generations (additional clock and data-size analyses), sampling every 2,500 or 5,000, respectively. 10% of the samples (corresponding to 1,000 sampled trees) were excluded before analysis as a burn-in. Stationarity was assessed using Tracer v1.6 (Rambaut et al., 2014), an application for visualizing MCMC traces. A maximum clade credibility tree was generated for each analysis using TreeAnnotator v1.7.5 assuming median node heights and a posterior probability limit of 0.5.

### Analysis of posterior distributions and maximum clade credibility trees

We analyzed each combination of the four possible simulation/estimation cases (Yule:Strict, Yule:UCLN, BD:Strict, and BD:UCLN) and number of taxa (25 or 100). First, each distribution of trees was rescaled to the exact root height of the original tree using ape (Paradis, Claude & Strimmer, 2004). This was performed to remove any error associated with estimating overall molecular rates of evolution and the overall age of the tree, allowing us to focus specifically on effects of priors on relative branching patterns. Then, for each tree in the posterior, we estimated λ and *r* by maximum likelihood using the DDD package in R (Etienne & Haegeman, 2012; Etienne et al., 2012).

In addition, we produced lineage-through-time (LTT) plots for each replicate. The LTT plot of the maximum clade credibility tree produced from each analysis was plotted on the same graph as the original tree from which the data were simulated. Each plot, then, consists of LTT plots for the 10 original trees and consensus trees from the corresponding 10 posterior distributions.

## Results

**Figure 2:**
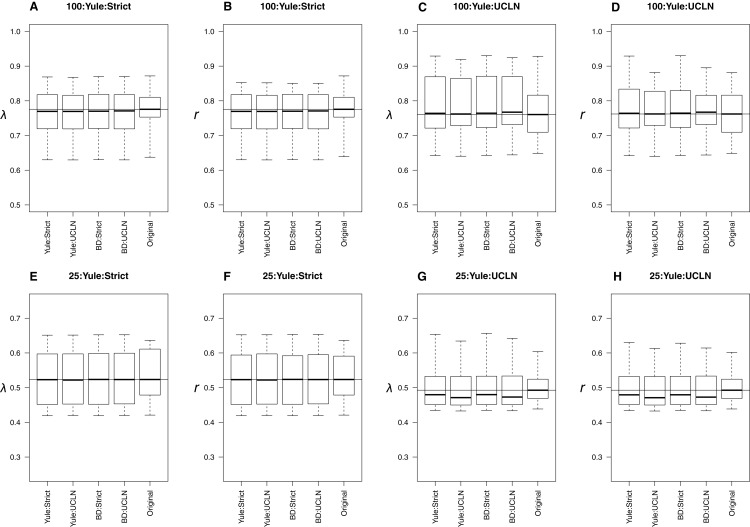
Yule simulations. The top row of plots (A–D) refers to the 100-taxa cases, whereas the bottom row (E–H) refers to the 25-taxa cases. Median estimates of λ or *r*, estimated from the 10 original trees, are used as data for each boxplot. The title of each subplot refers to the simulation conditions. Each combination of tree priors and molecular clocks under which trees are estimated is listed on the *x*-axis. The distribution of estimates from the original trees is also displayed. Parameter estimates are generally consistent with the original trees with slight deviations in some cases.

When original trees were simulated under a Yule process, all combinations of tree priors and clocks produced extremely similar estimates to the parameters estimated from trees on which data were simulated (Fig. 2). Distributions overlapped across all combinations of tree priors and molecular clocks. Slight deviations from simulated values are likely attributable to sampling error. The estimates of λ and *r* were consistently underestimated for the 25-taxa UCLN cases, providing evidence that the number of taxa is important when among-lineage rate heterogeneity is concerned. However, other preliminary trials did not show a consistent pattern of underestimation, suggesting that this pattern results from the 10 trees initially selected for simulation and not a systematic bias. LTT plots of maximum clade credibility trees indicated that the estimated trees generally coincide with the original trees, though the Yule:UCLN case showed greater discordance at nodes deeper in the tree for a small number of replicates (Fig. S1). This is not surprising given the difficulty of estimating nodes deep in the tree, and it also helps explain the discrepancy described above.

**Figure 3:**
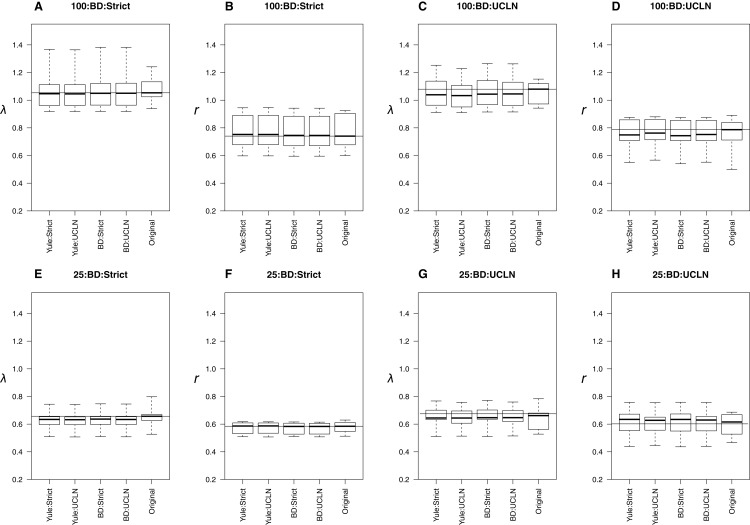
Birth-death simulations. The top row of plots (A–D) refers to the 100-taxa cases, whereas the bottom row (E–H) refers to the 25-taxa cases. The median estimates of λ or *r*, estimated from the 10 original trees, are used as data for each boxplot. The title of each subplot refers to the simulation conditions. Each combination of tree priors and molecular clocks under which trees are estimated is listed on the *x*-axis. The distribution of estimates from the original trees is also displayed. Parameter estimates are highly congruent with the original trees under each set of simulation conditions.

When trees were simulated under a BD process, estimates were also generally concordant with the original trees. Medians were nearly identical among many simulation conditions (Fig. 3), though parameters were underestimated in the UCLN cases. This discrepancy was either reduced or did not appear to be present in cases assuming a strict clock. LTT plots revealed that maximum clade credibility trees were, again, approximately equivalent to the original. There were some exceptions, again in the deep nodes of the trees, though these did not drastically affect parameter estimation (Fig. 3). As in the Yule cases, there were no discernable tendencies for parameter estimates to be consistently over or underestimated relative to the simulated trees in preliminary analyses. However, estimates of λ are biased downward, sometimes drastically. For the 25-taxa cases, λ estimates are close to *r*, even though they ought to be 2*r* with ε = 0.5. We hypothesize that estimates of λ should approach 2*r* as the number of taxa increases. To investigate, we performed additional simulations, as described above, but with 50, 75, and 125 taxa. Estimates of λ increase with the number of taxa but are still reduced (Fig. S3).

**Figure 4:**
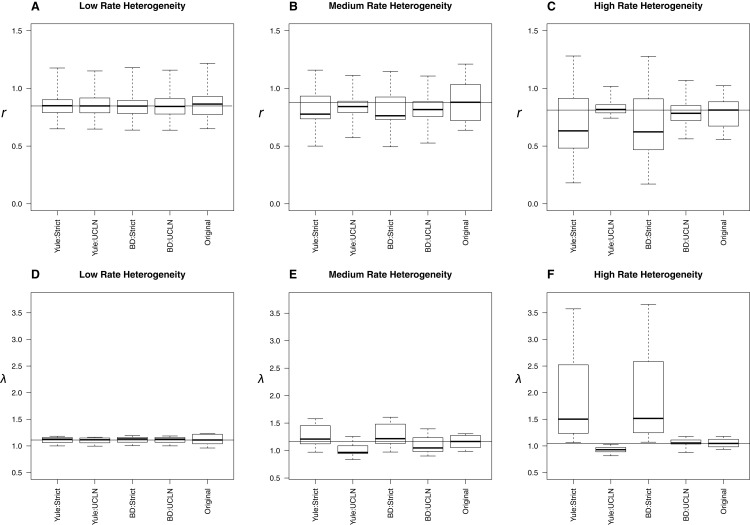
Effect of substitution rate heterogeneity on diversification rate estimates. Three simulated datasets with low (A, D), medium (B, E), and high (C, F) substitution rate heterogeneity (see Materials and Methods) are displayed. Parameter estimates agree with simulated data in the low heterogeneity case across all combinations of priors. The deviation of estimates from the original trees increases as the amount of heterogeneity increases. The effect is most pronounced in the “high” case, where the use of a strict molecular clock fails to capture heterogeneity and produces substantially different estimates. An uncorrelated log-normal molecular clock produces reasonable estimates in all cases.

With low, medium, and high among-lineage substitution rate heterogeneity, assumptions about molecular rates can have substantial impact on parameter estimates (Fig. 4). With low rate heterogeneity, estimates of λ and *r* are similar to the original trees, but the discordance increases dramatically as the variance in rates among lineages increases. Trees estimated using an UCLN clock appear to suffer the least, especially when estimated under the simulation conditions (BD:UCLN). This effect is most dramatic in the high rate heterogeneity simulations, where the assumption of a tree-wide constant substitution rate can lead to substantially discordant estimates of both λ and *r*. Further analysis of each of these simulation conditions indicates a deviation from a strict clock, as evidenced by posterior estimates of the coefficient of variation from BEAST on the simulated datasets (95% HPD, low rate hetereogeneity: [0.157–0.1948]; medium rate heterogeneity: [0.2508–1.1833]; high rate heterogeneity: [0.2402–2.6596]).

## Discussion

The goal of this study is to determine the impact the choice of tree prior and molecular clock have on the estimation of diversification rates. We focused our efforts on estimating λ, the rate of lineage accumulation, and *r*, the net diversification rate, under all combinations of two tree priors (Yule and BD) and two flavors of molecular clocks (strict and UCLN). These parameters were selected for investigation because estimating the relative extinction rate (ε) alone is known to be difficult, and estimates of this parameter have larger uncertainty (Nee et al., 1994). Estimating the net diversification rate still provides insight into the effect of extinction across the phylogeny while facilitating a meaningful comparison among simulation conditions. We found that the combination of tree prior and clock did not substantially impact diversification parameter estimates. Across our simulation conditions, parameters from trees estimated under all combinations of tree priors and clocks were concordant with parameter estimates produced from the trees on which nucleotide data were simulated. However, the fact that estimates of λ are biased downward, sometimes drastically, suggests that estimates of λ may be incorrect when trees are estimated assuming no extinction.

The simulations involving low, medium, and high among-lineage substitution rate heterogeneity revealed that it is possible for the choice of clock to have a substantial impact on parameter estimates (Fig. 4). Trees estimated using an UCLN clock appear to suffer the least, especially when estimated under the simulation conditions (BD:UCLN). This effect is most dramatic in the high rate heterogeneity simulations, where the assumption of a tree-wide constant substitution rate can lead to substantially discordant estimates of both λ and *r*. At the same time, investigators could easily avoid errors associated with using a strict clock by testing for rate heterogeneity in their sequence data.

The assumption of a single rate of evolution across a tree is often violated and can severely impair phylogenetic estimation (Shavit et al., 2007; Penny, 2013). This study assumed rates with a modest amount of heterogeneity, and it appears that a strict clock produces reasonable results in the face of this violation. In other words, a dataset with a small to moderate amount of heterogeneity may have rates that are reasonably captured by a single, global rate. However, it may not be known a priori whether a dataset has disparate rates of evolution among lineages. It would be advisable, then, to assume a clock model that has the potential to model heterogeneity more accurately, and this is partially why the UCLN relaxed clock has seen such widespread use and success in systematic analyses (Drummond et al., 2006). Furthermore, should rates of evolution be extreme among some lineages, it would make sense to attempt to capture any heterogeneity using appropriate priors as opposed to assuming it is absent. Rate homogeneity among lineages, or the absence of a clock altogether, may represent a poor prior given our current understanding of molecular biological processes (Drummond et al., 2006).

There are several caveats to this simulation study. First, our original trees are fully resolved, and nucleotide sequence data are simulated under parameters estimated from a quickly evolving nuclear intron. This implies that there will be a large number of phylogenetically informative sites per individual. Therefore, these trees will be easier to estimate than those that lack signal and/or contain unresolved nodes. Second, there is no extreme rate heterogeneity among lineages. Third, the datasets only contain 25 and 100 taxa, each with only 5,000 bp of nucleotide sequence data, following the protocol of Wertheim & Sanderson (2011). Datasets of this size are considered modest in the current era of high-throughput sequencing, where the generation of hundreds of thousands or millions of base pairs of sequence per sample is possible. More sequence data can lead to more accurate phylogenies, which improves parameter estimates at the expense of computational speed. It is also reasonable to assume that some systems may be best explained through more complex models, such as models that specifically assume multiple, independent diversification rates across a dataset (Alfaro et al., 2009; Rabosky, 2014). Our analyses only assume a single rate of diversification, and this assumption may be violated in larger datasets with greater levels of taxonomic divergence. Furthermore, there are families of models that assume shifts in speciation rates across phylogenies which could be considered (Steel & McKenzie, 2001). Such models can be fit to identify diversification rate heterogeneity and, therefore, estimate diversification rates more accurately under complex scenarios. Finally, by fixing root age, we control for known sources of estimation error that have to do with calibrating molecular evolution when reconstructing time trees. Careful attention to calibrations is essential to obtaining diversification rates in units that are meaningful. We reinforce that it is important to select among models in order to produce accurate, interpretable results for each dataset.

## Conclusions

It appears that reasonable parameter estimates can often be achieved regardless of the prior used for phylogenetic tree shape. Among the cases that we simulated, either choice of tree prior appears to capture the underlying branching process; the same holds for molecular clocks with low among-lineage rate heterogeneity. Even in cases with excessive among-lineage rate heterogeneity, it is generally true that existing methods are able to detect and account for that rate variation. Overall, we find that the choice of tree prior and molecular clock has relatively little impact on the estimation of diversification rates.

## Supplemental Information

10.7717/peerj.6334/supp-1 Supplemental Information 1 Lineage-through-time plots. The y-axis of each plot is natural-log transformed. Rows refer to conditions under which original trees are simulated, and columns refer to conditions under which trees are estimated. Thick gray lines represent the original trees and are, therefore, identical across each row of plots. Thin dark lines refer to the maximum clade credibility trees summarized from the posterior distribution of trees under the specified combination of tree prior and molecular clock. There is a significant amount of concordance, indicative of accurate phylogenetic estimation, though some discordance (indicated by non-overlapping lines) is revealed.

10.7717/peerj.6334/supp-2 Supplemental Information 2 The effect of data size on diversification rate estimates. Estimates of net diversification rate approach the values of the original simulations as the size of the dataset increases, especially for the combination of tree prior and molecular clock under which the data were simulated (i.e., BD:UCLN).

10.7717/peerj.6334/supp-3 Supplemental Information 3 The effect of the number of taxa on estimates of net diversification rate and speciation rate. The x-axis (125, 75, or 50) corresponds to the number of taxa, and the y-axis corresponds to the value of the parameter estimated (*λ* or *r*). Since posterior distributions were generated under a birth-death process with the relative extinction rate equal to 0.5, estimates of *λ* should approach 2*r*. While the difference from the expectation improves with the number of taxa, the discrepancy can be attributed to a combination of sample size and model misspecification (i.e., estimating *λ* assuming a Yule process when the generating model was Birth-Death).

## Additional Information and Declarations

### Data Availability

The following information was supplied regarding data availability: Code is available at GitHub: https://github.com/bricesarver/prior_simulation_study.

## References

[ref-1] Alfaro ME, Santini F, Brock C, Alamillo H, Dornburg A, Rabosky DL, Carnevale G, Harmon LJ (2009). Nine exceptional radiations plus high turnover explain species diversity in jawed vertebrates. Proceedings of the National Academy of Sciences of the United States of America.

[ref-2] Barraclough TG, Nee S (2001). Phylogenetics and speciation. Trends in Ecology & Evolution.

[ref-3] Brown JW (2014). https://github.com/josephwb/BEASTifier.

[ref-4] Brown RP, Yang Z (2010). Bayesian dating of shallow phylogenies with a relaxed clock. Systematic Biology.

[ref-5] Condamine FL, Nagalingum NS, Marshall CR, Morlon H (2015). Origin and diversification of living cycads: a cautionary tale on the impact of the branching process prior in Bayesian molecular dating. BMC Evolutionary Biology.

[ref-6] Cusimano N, Renner SS (2010). Slowdowns in diversification rates from real phylogenies may not be real. Systematic Biology.

[ref-7] Drummond AJ, Ho SYW, Phillips MJ, Rambaut A (2006). Relaxed phylogenetics and dating with confidence. PLOS Biology.

[ref-8] Drummond AJ, Nicholls GK, Rodrigo AG, Solomon W (2002). Estimating mutation parameters, population history and genealogy simultaneously from temporally spaced sequence data. Genetics.

[ref-9] Drummond AJ, Rambaut A, Shapiro B, Pybus OG (2005). Bayesian coalescent inference of past population dynamics from molecular sequences. Molecular Biology and Evolution.

[ref-10] Drummond AJ, Suchard MA, Xie D, Rambaut A (2012). Bayesian phylogenetics with BEAUti and the BEAST 1.7. Molecular Biology and Evolution.

[ref-11] Duchêne DA, Hua X, Bromham L (2017). Phylogenetic estimates of diversification rate are affected by molecular rate variation. Journal of Evolutionary Biology.

[ref-12] Etienne RS, Haegeman B (2012). A conceptual and statistical framework for adaptive radiations with a key role for diversity dependence. American Naturalist.

[ref-13] Etienne RS, Haegeman B, Stadler T, Aze T, Pearson PN, Purvis A, Phillimore AB (2012). Diversity-dependence brings molecular phylogenies closer to agreement with the fossil record. Proceedings of the Royal Society B: Biological Sciences.

[ref-14] Felsenstein J (1985). Phylogenies and the comparative method. American Naturalist.

[ref-15] FitzJohn RG (2010). Quantitative traits and diversification. Systematic Biology.

[ref-16] FitzJohn RG, Maddison WP, Otto SP (2009). Estimating trait-dependent speciation and extinction rates from incompletely resolved phylogenies. Systematic Biology.

[ref-17] Gelman A, Carlin JB, Stern HS, Rubin DB (1995). Bayesian data analysis.

[ref-18] Gernhard T (2008). The conditioned reconstructed process. Journal of Theoretical Biology.

[ref-19] Harmon LJ (2018). Phylogenetic comparative methods: learning from trees. https://github.com/lukejharmon/pcm.

[ref-20] Harvey PH, Pagel MD (1991). The comparative method in evolutionary biology.

[ref-21] Ho SYW, Phillips MJ, Drummond AJ, Cooper A (2005). Accuracy of rate estimation using relaxed-clock models with a critical focus on the early metazoan radiation. Molecular Biology and Evolution.

[ref-22] Höhna S (2014). Likelihood inference of non-constant diversification rates with incomplete taxon sampling. PLOS ONE.

[ref-23] Huelsenbeck JP, Ronquist F, Nielsen R, Bollback JP (2001). Bayesian inference of phylogeny and its impact on evolutionary biology. Science.

[ref-24] Kendall DG (1948). On the generalized “Birth-and-Death” process. Annals of Mathematical Statistics.

[ref-25] Kingman JFC (1982). The coalescent. Stochastic Processes and their Applications.

[ref-26] Lepage T, Bryant D, Philippe H, Lartillot N (2007). A general comparison of relaxed molecular clock models. Molecular Biology and Evolution.

[ref-27] Maddison WP, Midford PE, Otto SP (2007). Estimating a binary character’s effect on speciation and extinction. Systematic Biology.

[ref-28] Magallón S, Sanderson MJ (2001). Absolute diversification rates in angiosperm clades. Evolution.

[ref-29] Marin J, Hedges SB (2018). Undersampling genomes has biased time and rate estimates throughout the tree of life. Molecular Biology and Evolution.

[ref-30] Moore BR, Höhna S, May MR, Rannala B, Huelsenbeck JP (2016). Critically evaluating the theory and performance of Bayesian analysis of macroevolutionary mixtures. Proceedings of the National Academy of Sciences of the United States of America.

[ref-31] Nee S (2006). Birth-death models in macroevolution. Annual Review of Ecology, Evolution, and Systematics.

[ref-32] Nee S, Holmes EC, May RM, Harvey PH (1994). Extinction rates can be estimated from molecular phylogenies. Philosophical Transactions of the Royal Society B: Biological Sciences.

[ref-33] Nee S, May RM, Harvey PH (1994). The reconstructed evolutionary process. Philosophical Transactions of the Royal Society B: Biological Sciences.

[ref-34] Nee S, Mooers AO, Harvey PH (1992). Tempo and mode of evolution revealed from molecular phylogenies. Proceedings of the National Academy of Sciences of the United States of America.

[ref-35] O’Meara BC (2012). Evolutionary inferences from phylogenies: a review of methods. Annual Review of Ecology, Evolution, and Systematics.

[ref-36] Paradis E, Claude J, Strimmer K (2004). APE: analyses of Phylogenetics and Evolution in R language. Bioinformatics.

[ref-37] Pennell MW, Sarver BAJ, Harmon LJ (2012). Trees of unusual size: biased inference of early bursts from large molecular phylogenies. PLOS ONE.

[ref-38] Penny D (2013). Rewriting evolution–“been there, done that”. Genome Biology and Evolution.

[ref-39] Pybus OG, Harvey PH (2000). Testing macro-evolutionary models using incomplete molecular phylogenies. Proceedings of the Royal Society of London. Series B: Biological Sciences.

[ref-40] Pyron RA, Burbrink FT (2013). Phylogenetic estimates of speciation and extinction rates for testing ecological and evolutionary hypotheses. Trends in Ecology & Evolution.

[ref-41] R Development Core Team (2018). R: a language and environment for statistical computing.

[ref-42] Rabosky DL (2010). Extinction rates should not be estimated from molecular phylogenies. Evolution.

[ref-43] Rabosky DL (2014). Automatic detection of key innovations, rate shifts, and diversity-dependence on phylogenetic trees. PLOS ONE.

[ref-44] Rabosky DL (2016). Challenges in the estimation of extinction from molecular phylogenies: A response to Beaulieu and O’Meara. Evolution.

[ref-45] Rambaut A, Grassly NC (1997). Seq-Gen: an application for the Monte Carlo simulation of DNA sequence evolution along phylogenetic trees. Bioinformatics.

[ref-46] Rambaut A, Suchard MA, Xie D, Drummond AJ (2014). http://beast.community/tracer.

[ref-47] Revell L, Harmon L, Glor R (2005). Under-parameterized model of sequence evolution leads to bias in the estimation of diversification rates from molecular phylogenies. Systematic Biology.

[ref-48] Ricklefs RE (2007). Estimating diversification rates from phylogenetic information. Trends in Ecology & Evolution.

[ref-49] Ritchie AM, Lo N, Ho SYW (2017). The impact of the tree prior on molecular dating of data sets containing a mixture of inter- and intraspecies sampling. Systematic Biology.

[ref-50] Ronquist F, Teslenko M, Van Der Mark P, Ayres DL, Darling A, Höhna S, Larget B, Liu L, Suchard MA, Huelsenbeck JP (2012). MrBayes 3.2: efficient Bayesian phylogenetic inference and model choice across a large model space. Systematic Biology.

[ref-51] Sanderson MJ (2003). r8s: inferring absolute rates of molecular evolution and divergence times in the absence of a molecular clock. Bioinformatics.

[ref-52] Schluter D, Pennell MW (2017). Speciation gradients and the distribution of biodiversity. Nature.

[ref-53] Shavit L, Penny D, Hendy MD, Holland BR (2007). The problem of rooting rapid radiations. Molecular Biology and Evolution.

[ref-54] Smith SA, O’Meara BC (2012). treePL: divergence time estimation using penalized likelihood for large phylogenies. Bioinformatics.

[ref-55] Stadler T (2013). How can we improve accuracy of macroevolutionary rate estimates?. Systematic Biology.

[ref-56] Steel M, McKenzie A (2001). Properties of phylogenetic trees generated by Yule-type speciation models. Mathematical Biosciences.

[ref-57] Weisrock DW, Harmon LJ, Larson A (2005). Resolving deep phylogenetic relationships in salamanders: analyses of mitochondrial and nuclear genomic data. Systematic Biology.

[ref-58] Wertheim JO, Sanderson MJ (2011). Estimating diversification rates: how useful are divergence times?. Evolution.

[ref-59] Yule GU (1925). A mathematical theory of evolution, based on the conclusions of Dr. J. C. Willis, F.R.S. Philosophical Transactions of the Royal Society B: Biological Sciences.

[ref-60] Zuckerkandl E, Pauling LB, Kasha M, Pullman B (1962). Molecular disease, evolution, and genic heterogeneity. Horizons in Biochemistry.

