# Supplementary figures and images for: The choice of tree prior and molecular clock does not substantially affect phylogenetic inferences of diversification rates
Source: PeerJ. 2019 Mar 13;7:e6334. doi: 10.7717/peerj.6334 (PMC6421065; doi:10.7717/peerj.6334)

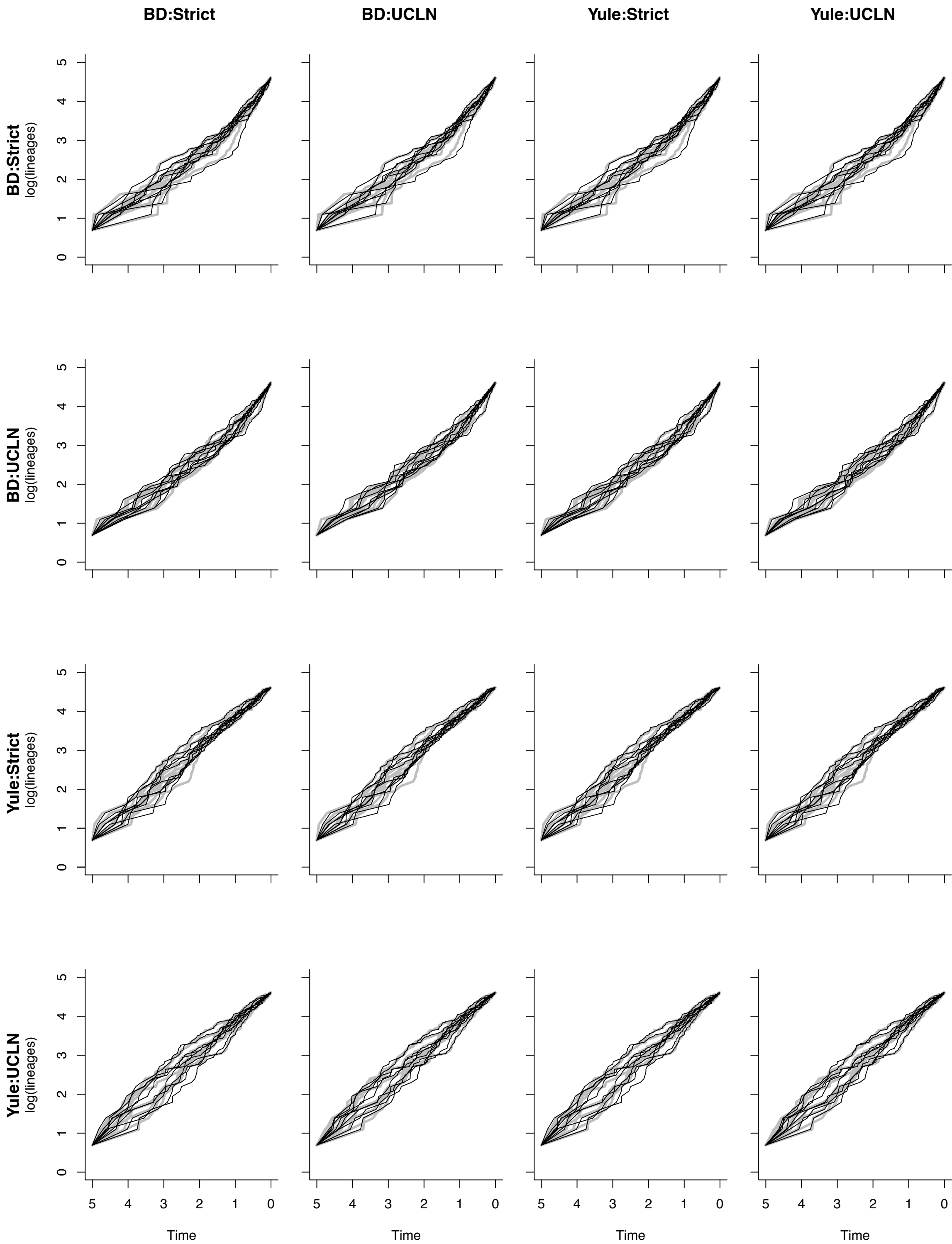

Supplement: Supplemental Information 1 — The y-axis of each plot is natural-log transformed. Rows refer to conditions under which original trees are simulated, and columns refer to conditions under which trees are estimated. Thick gray lines represent the original trees and are, therefore, identical across each row of plots. Thin dark lines refer to the maximum clade credibility trees summarized from the posterior distribution of trees under the specified combination of tree prior and molecular clock. There is a significant amount of concordance, indicative of accurate phylogenetic estimation, though some discordance (indicated by non-overlapping lines) is revealed. [file peerj-07-6334-s001.pdf]

2500bp

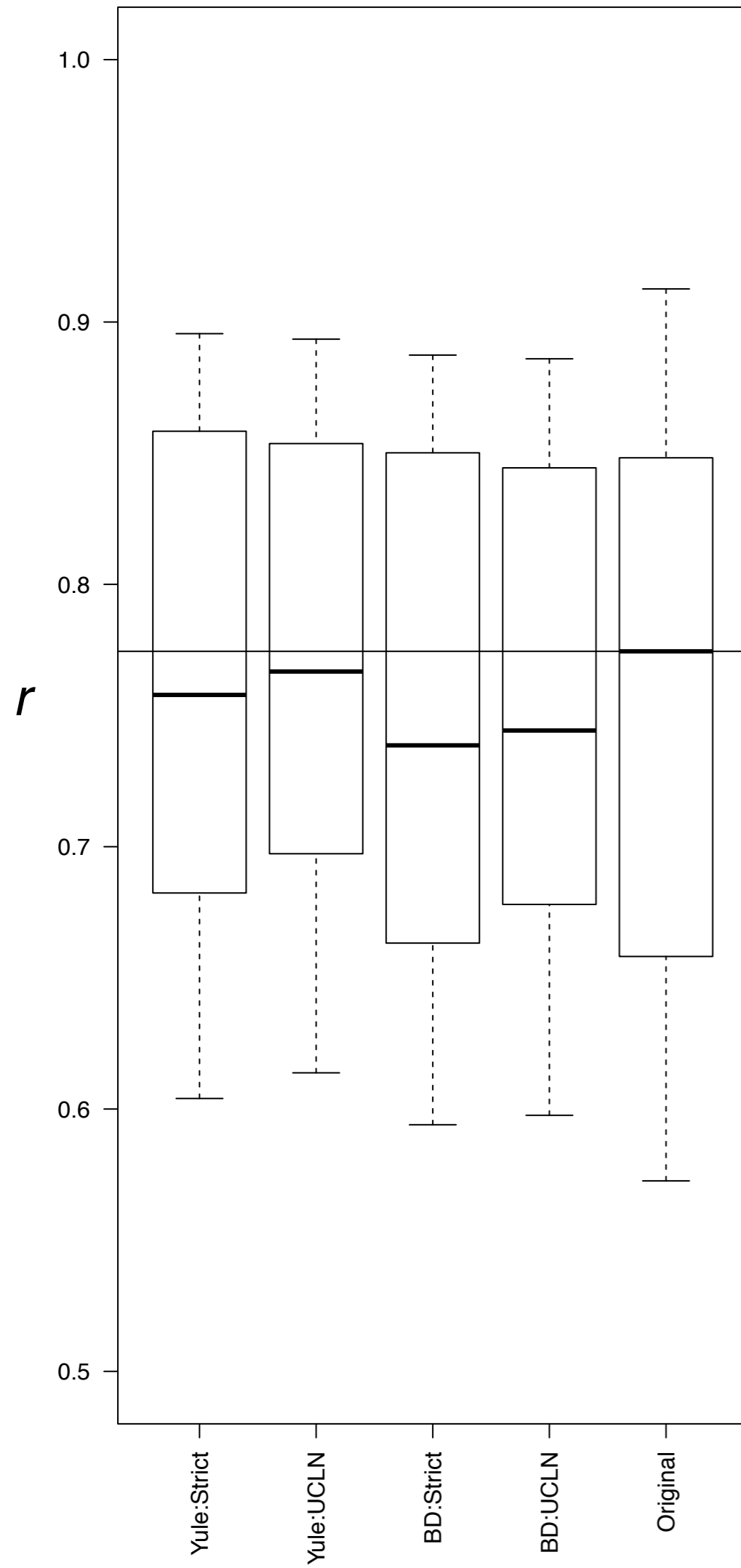

5000bp

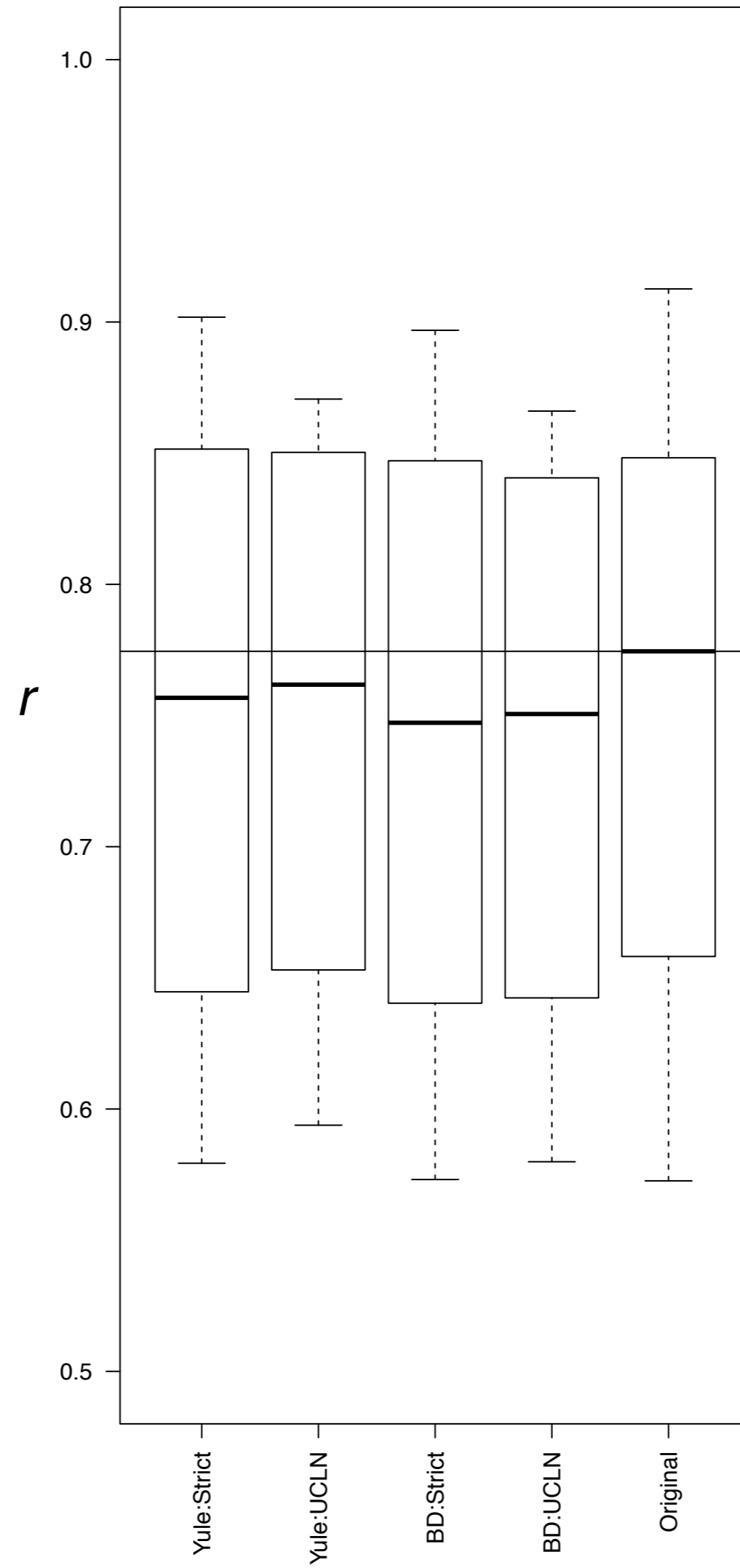

10000bp

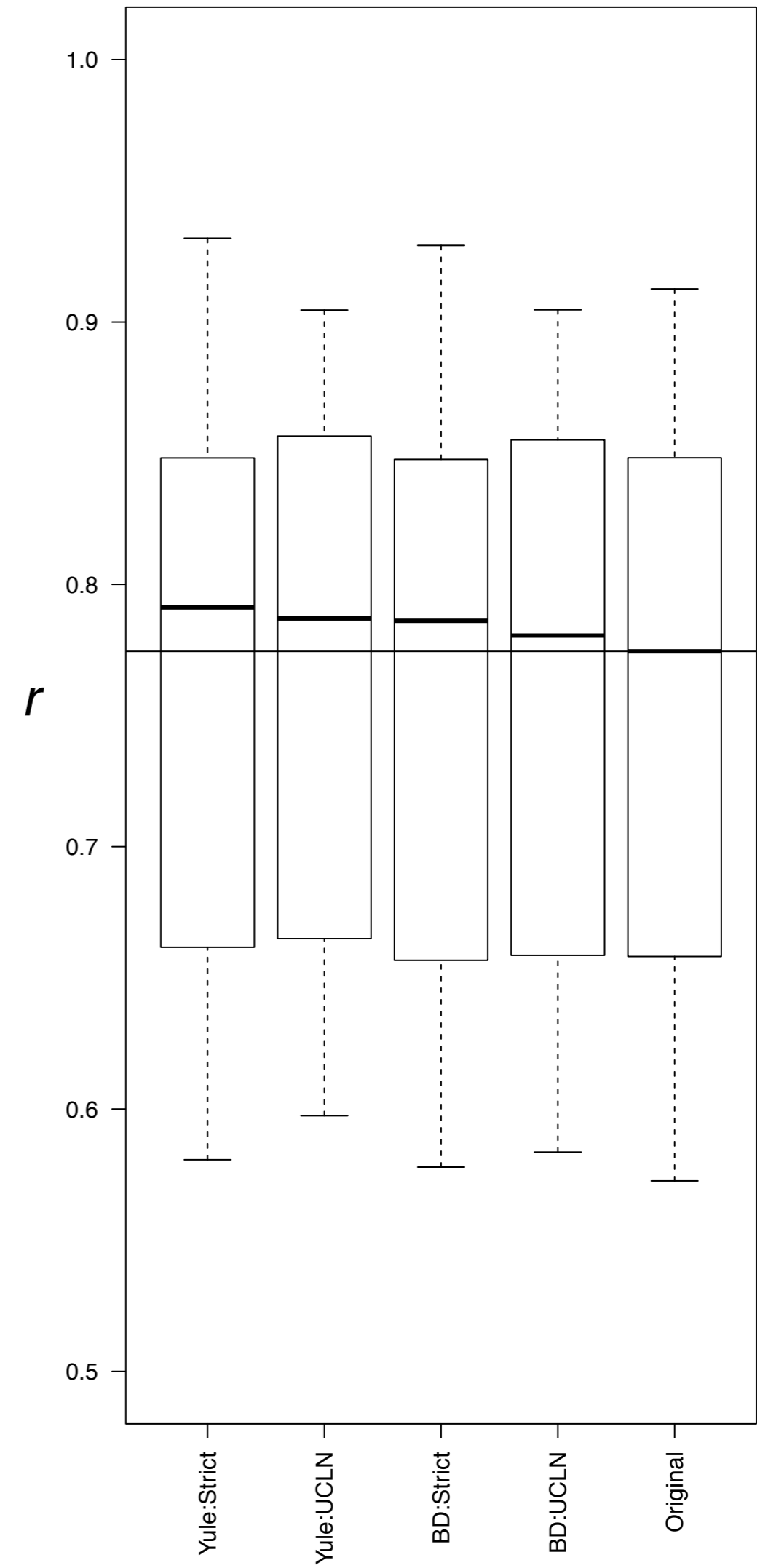

Supplement: Supplemental Information 2 — Estimates of net diversification rate approach the values of the original simulations as the size of the dataset increases, especially for the combination of tree prior and molecular clock under which the data were simulated (i.e., BD:UCLN). [file peerj-07-6334-s002.pdf]

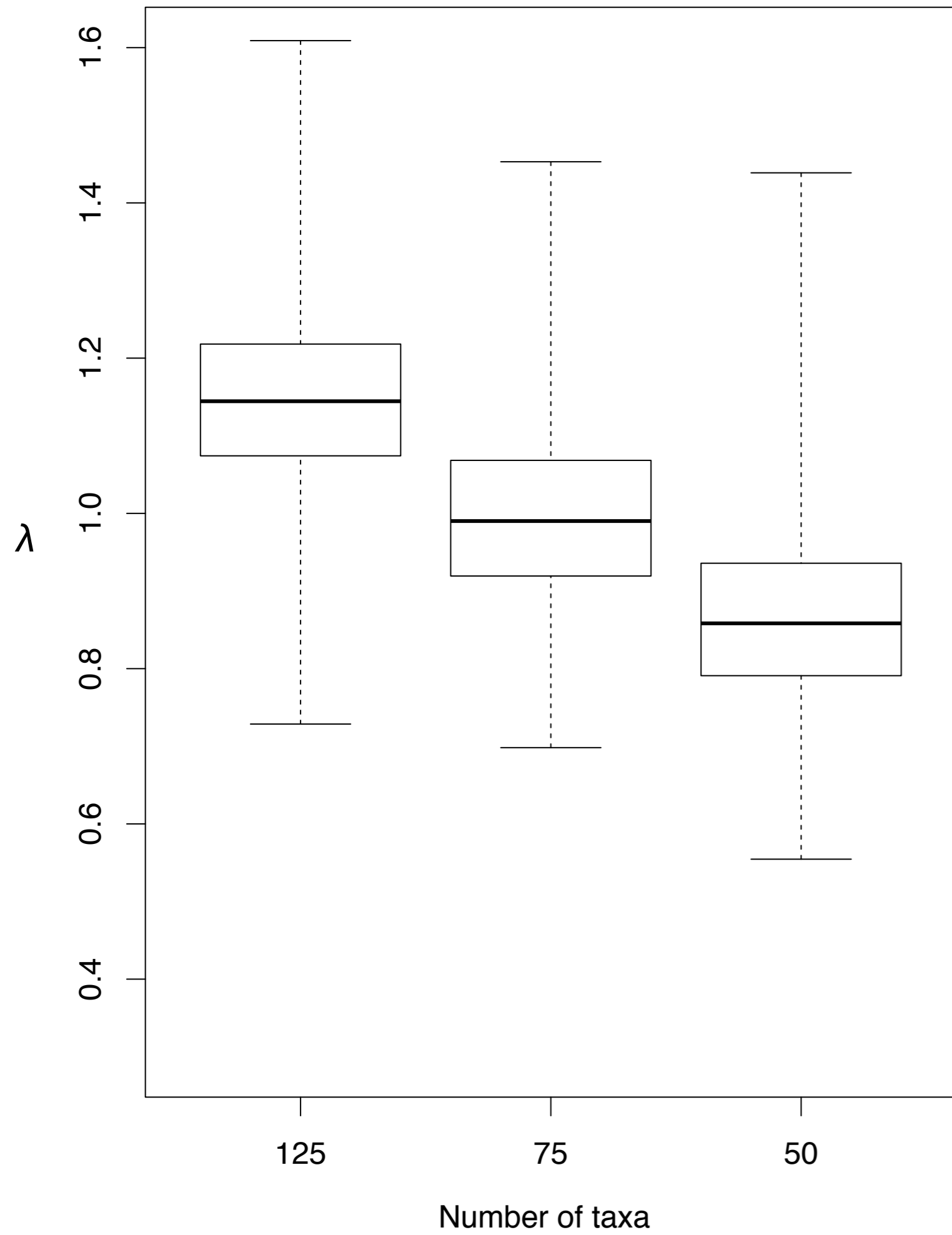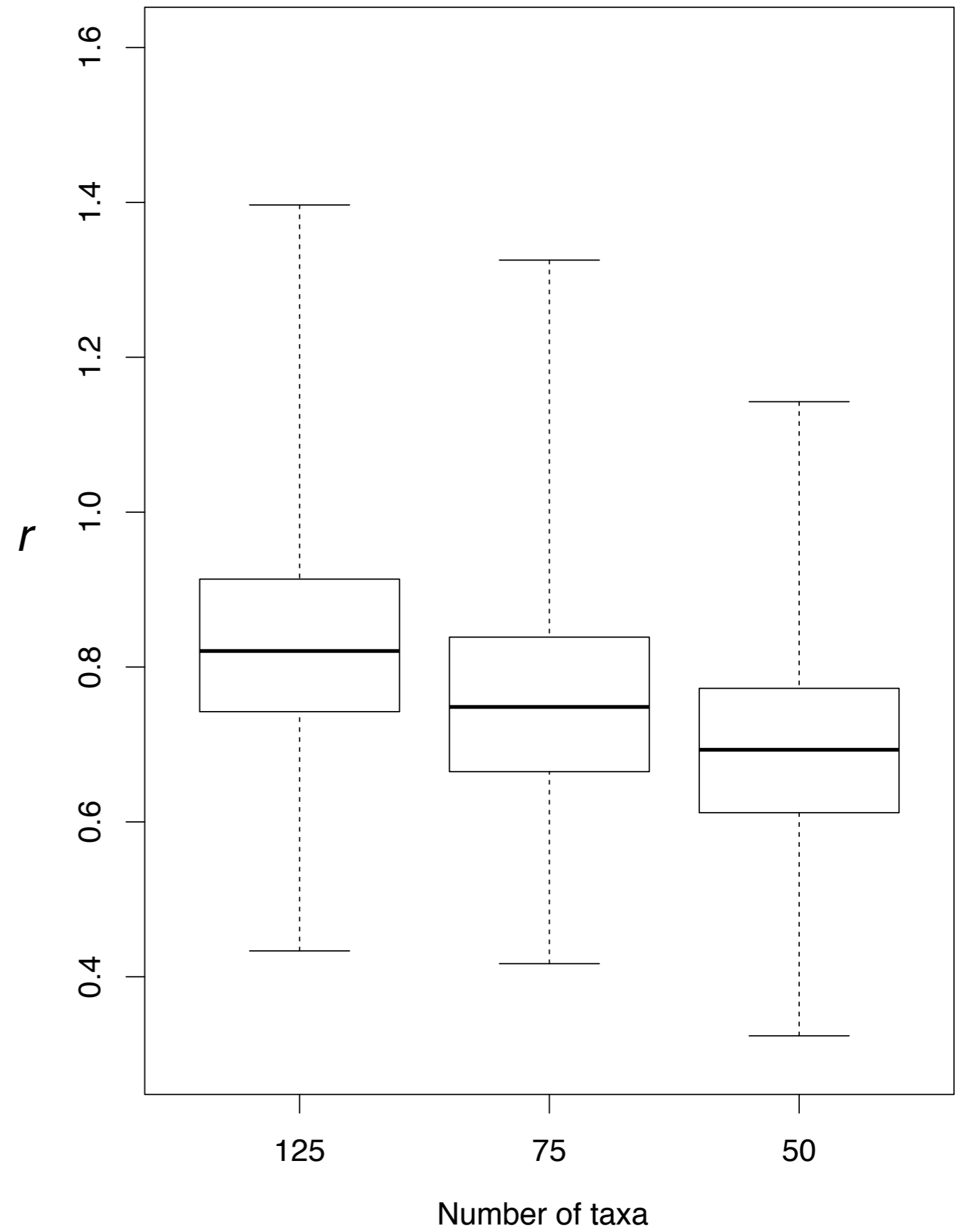

Supplement: Supplemental Information 3 — The x-axis (125, 75, or 50) corresponds to the number of taxa, and the y-axis corresponds to the value of the parameter estimated (λ or r). Since posterior distributions were generated under a birth-death process with the relative extinction rate equal to 0.5, estimates of λ should approach 2r. While the difference from the expectation improves with the number of taxa, the discrepancy can be attributed to a combination of sample size and model misspecification (i.e., estimating λ assuming a Yule process when the generating model was Birth-Death). [file peerj-07-6334-s003.pdf]
